# Histopathological data of iron and calcium in the mouse lung after asbestos exposure

**DOI:** 10.1016/j.dib.2016.01.026

**Published:** 2016-01-21

**Authors:** Elisa Trevisan, Giuliano Zabucchi, Lorella Pascolo, Ernesto Pascotto, Claudia Casarsa, Monica Lucattelli, Giuseppe Lungarella, Eleonora Cavarra, Barbara Bartalesi, Marina Zweyer, Violetta Borelli

**Affiliations:** a Department of Life Science, University of Trieste, 34127 Trieste, Italy; b Institute for Maternal and Child Health, IRCCS Burlo Garofolo, 34137 Trieste, Italy; c Servizio Diagnostica Veterinaria, University of Udine, 33100 Udine, Italy; d Department of Life Science, Section of Experimental Pathology, University of Siena, 53100 Siena, Italy; e Dipartimento Universitario Clinico di Scienze Mediche, Chirurgiche e della Salute, Università di Trieste, 34149 Trieste, Italy

**Keywords:** Asbestos, Animal model, Iron, Calcium, Histochemistry, Histopathology

## Abstract

This data article contains data related to the research article entitled, “Synchrotron X-ray microscopy reveals early calcium and iron interaction with crocidolite fibers in the lung of exposed mice” [1]. Asbestos fibers disrupt iron homeostasis in the human and mouse lung, leading to the deposition of iron (Fe) onto longer asbestos fibers which forms asbestos bodies (AB) [2]. Similar to Fe, calcium (Ca) is also deposited in the coats of the AB. This article presents data on iron and calcium in the mouse lung after asbestos exposure detected by histochemical evaluation.

**Specifications Table**

**Table:** 

Subject area	*Biology*
More specific subject area	*Histopathology/histochemistry*
Type of data	*Image (optical microscopy), text file and graph.*
How data was acquired	*Microscope (Orthoplan microscope, equipped with a JVC TK-C1381EG digital color video camera), automated cell counting (Coulter, Miami, FL, USA).*
Data format	*Raw and analyzed*
Experimental factors	*Pathogen-free 6–8 week old male C57Bl/6 mice were instilled intratracheally with 100 µg of crocidolite asbestos in sterile saline or saline only (control mice).*
Experimental features	*One month after saline or asbestos instillation, mice were sacrificed and examined for bronchoalveolar lavage fluid, lung pathology and lung iron and calcium distribution.*
Data source location	*Siena and Trieste, Italy*
Data accessibility	*The data are within this paper.*

**Value of the data**

• Asbestos fibers in the lung may be associated with crystalline deposits of calcium.
• Histochemical evaluation of lung calcium after asbestos exposure has never been performed.
• We present data about the deposition of calcium in lung tissue in a murine model of asbestos exposure employing the classic histological method for the detection of calcium deposits in animals: the Von Kossa staining technique.
• The data indicate that a very low amount of calcium deposition is detected using this technique.
• The histochemistry data shown here can be useful for any further analysis employing other methods of detection of calcium, to better evaluate calcium deposition and its possible correlation with the asbestos induced inflammatory response.

## 1 Data

Here we describe the histopathological and histochemical changes in lung tissues of mice following asbestos exposure.

**Fig. 1:**
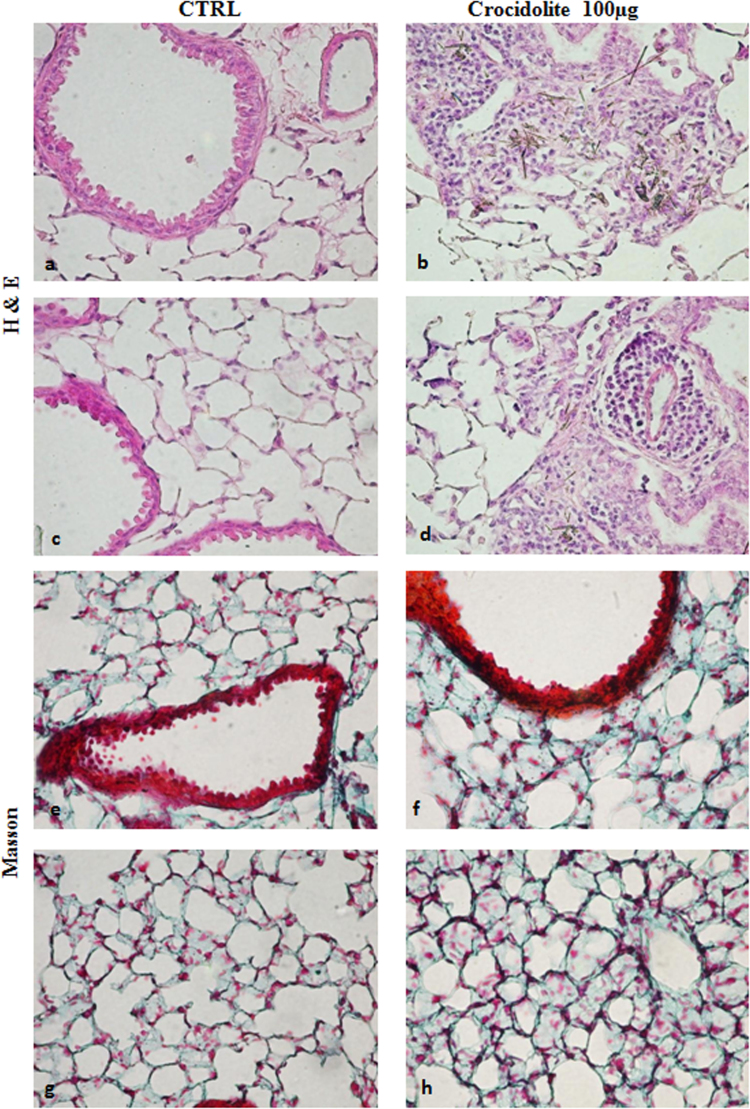
Lung inflammation and fibrosis. Lung inflammation. Representative images of H&E-stained lung sections: (a and c) saline exposure; (b and d) crocidolite (100 µg) exposure. Lung inflammation increases after crocidolite exposure. Original magnification: 400×. Lung fibrosis. Representative images of Masson׳s trichrome-stained lung sections: (e and g) saline exposure; (f and h) crocidolite exposure. The fibrotic response (collagen fibers stained blue) is higher after crocidolite exposure. Original magnification: 400×.

**Fig. 2:**
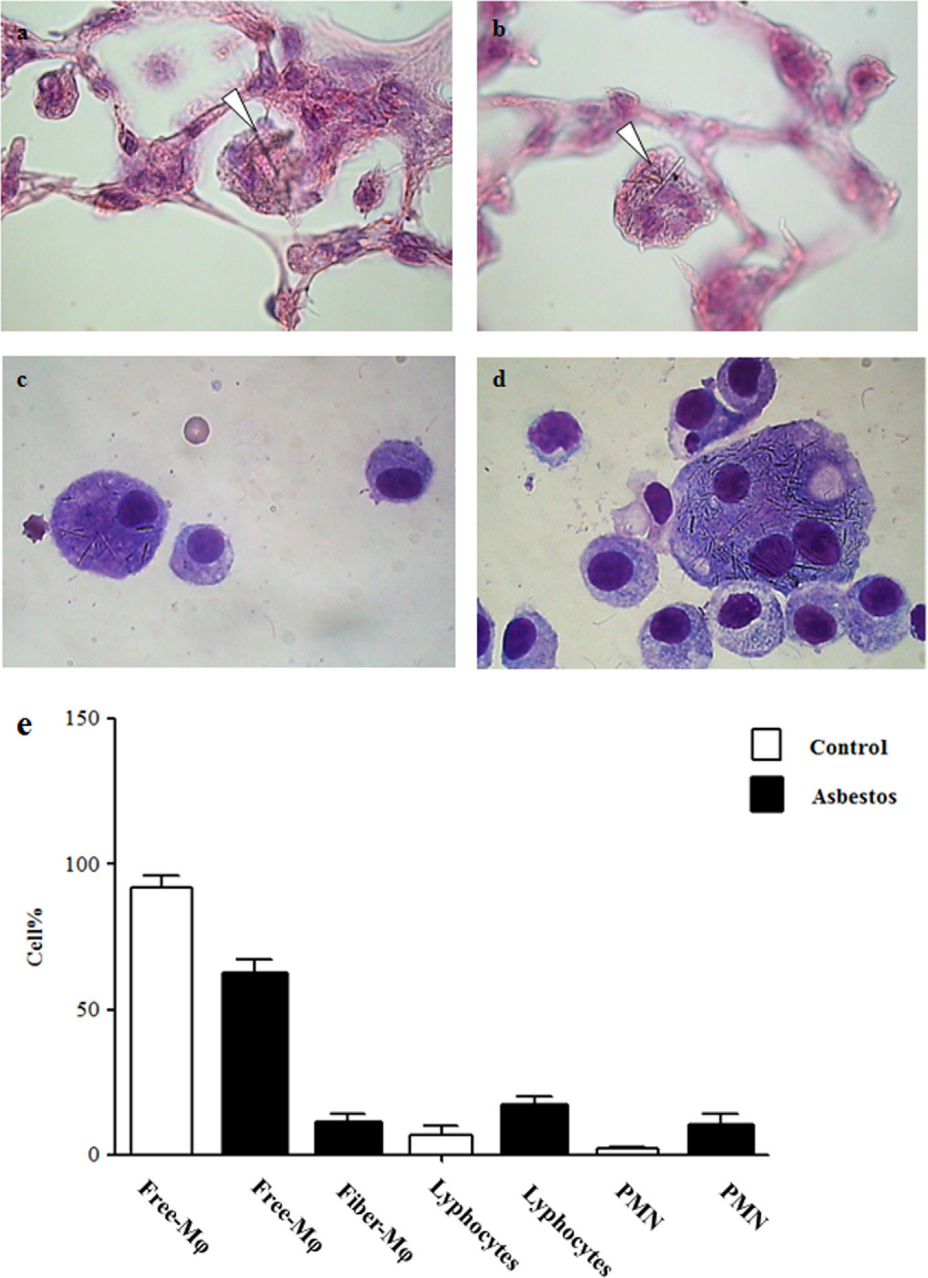
Lung inflammation. Panels a and b: representative images of H&E-stained lung sections. Multinucleated giant cells that appear to have ingested asbestos fibers (arrowheads). Original magnification: 400×. Panels c and d: representative images of cytospin preparations of BALF after staining with Diff-Quick. Original magnification: 1000×. Panel e: differential cell counts (%) in BALF samples from saline- (Control) and asbestos-exposed (Asbestos) mice at 1 month. Columns, mean; bars, ±SE; *, significantly different (*P*< 0.05) from control group.

Our data reveals that after 1 month of asbestos exposure, crocidolite fibers are observed most frequently in the distal bronchioles region (Fig. 1b and d) and that the retention of asbestos fibers is associated with an accumulation of inflammatory cells, predominantly macrophages, which appeared to have ingested asbestos fibers (Fig. 2a and b).

Data of bronchoalveolar lavage fluid analysis (Fig. 2c and d) reveals in exposed animals an increase in total cells and in the percentages of neutrophils and lymphocytes (Fig. 2e).

Increased collagen are observed in various regions of the lung of mice exposed to asbestos (Fig. 1e and g), compared to control animals exposed to saline (Fig. 1f and h).

**Fig. 3:**
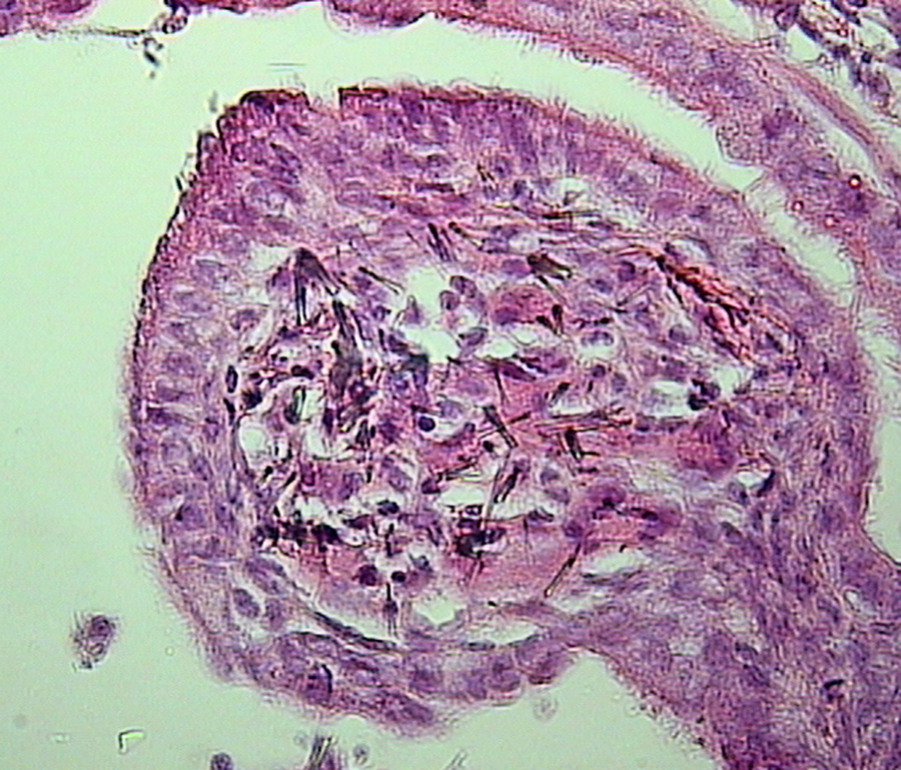
Hyperplastic lesion, characterized by stacked, multiple layers of epithelial cells lining alveolar ducts (original magnification ×400).

Focal atypical hyperplastic lesions located at or near terminal bronchioles are present in all the treated animals. The lesions are characterized by stacked, multiple layers of epithelial cells lining alveolar ducts or alveolar walls (Fig. 3).

**Fig. 4:**
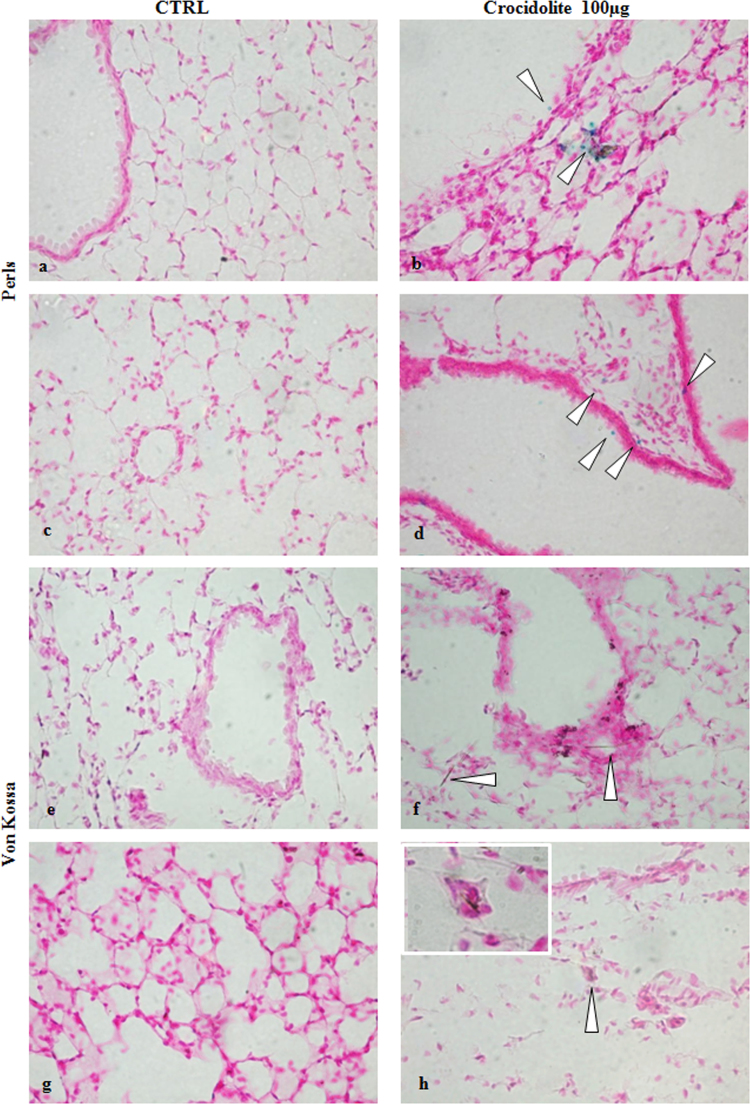
Iron and calcium deposition in the lung after crocidolite exposure. Iron lung positivity. Representative images of Perls׳ Prussian blue-stained lung sections: (a and c) saline exposure; (b and d) crocidolite (100 µg) exposure. Original magnification: 400×. Calcium lung positivity. Representative images of Von Kossa-stained lung sections: (e and g) saline exposure; (f and h) crocidolite exposure. Inset in panel h shows the simultaneous presence of crocidolite fiber and Von Kossa positive staining in a multinucleated macrophage free in the alveolar space. Arrows in figure f and h show the presence of crocidolite fibers. Original magnification: 400×.

Histological lung sections from saline exposed mice do not stain either for iron or for calcium (Fig. 4, panels a, c and e, g). One month after crocidolite instillation, very few iron positive macrophage-like cells are present in the alveolar region (Fig. 4, arrows in panels b and d).

Tissue analysis for calcium deposits reveals very weak positive signals, associated with macrophages and asbestos fibers deposition (Fig. 4, arrows in panels f and h and inset in h).

## 2 Experimental design, materials and methods

### 2.1 Crocidolite fibers

Analytical standard UICC samples of crocidolite asbestos were obtained from SPI-CHEM, West Chester, PA. The fiber suspension was prepared as described by Pascolo and colleagues [1].

### 2.2 Animals

C57Bl/6 male mice, a strain sensitive to asbestos-induced lung injury [3], were purchased from Charles River Laboratories (Calco, Italy). All experiments were performed at the Department of Life Sciences, Section of Experimental Pathology, of the University of Siena (Italy), according to the institutional guidelines of the animal ethics committee of the Italian government. The animals were treated humanely and with regard for alleviation of suffering. All animal experiments were conducted in conformity with the “Guiding Principles for Research Involving Animals and Human Beings” and the Local Ethics Committee of the University of Siena approved the protocols.

### 2.3 Asbestos exposure: intratracheal instillation of crocidolite

Mice were exposed to asbestos according to methods previously described by Adamson and Bowden [4]. Briefly, pathogen-free 6–8 week old male C57Bl/6 mice (*n*=12), were instilled intratracheally with 100 µg of crocidolite asbestos in sterile PBS (60 μl) while under light ether anesthesia. Control mice (*n*=12) received sterile PBS only. One month after saline or asbestos instillation, mice were sacrificed and examined for bronchoalveolar lavage fluid (BALF) (*n*=5), lung pathology and lung histological staining for iron and calcium (*n*=7).

### 2.4 BALF collection

With the aid of a peristaltic pump (P-1 Pharmacia), the lungs (*n*=5 for each group) were lavaged *in situ* three times with 0.6 ml of saline solution. The average fluid recovery was 95%. Total cell numbers were counted (Coulter, Miami, FL, USA) and differential cell counts were performed on cytospin preparations after staining with Diff-Quick (Medion Diagnostics, Dudingen, Switzerland).

### 2.5 Lung histopathology and histological staining for iron and calcium

One month after saline or asbestos instillation, mice (7 animals for each group) were sacrificed and the lungs were fixed intratracheally with formalin (5%) at a pressure of 20 cm H_2_O. The left lung of each mice was embedded in paraffin, sectioned to a thickness of 5 μm, and stained with hematoxylin and eosin (H&E) and Masson׳s trichrome.

Evaluation of inflammation. Mice from each treatment group (*n*=7/group) were evaluated through the analysis of at least five sections per mouse. Inflammation was determined by the amount and general appearance of infiltrating leukocytes (polymorphonuclear cells and macrophages) as seen by hematoxylin and eosin staining (Fig. 1a–d and Fig. 2a and b). Histopathological changes in the tissues were also evaluated taking into consideration fibrosis (Fig. 1e–h) and changes in alveolar epithelium (Fig. 3). Inflammation was also assessed from the changes in BALF׳s total and differential cell counts (Fig. 2c–e). A Perl׳s blue histochemical stain was used to reveal ferric iron (NovaUltra Prussian Blue Stain Kit, IHC WORLD, Woodstock, MD, USA) (Fig. 4a–d). Tissue staining for calcium was performed by Von Kossa, which stains calcium brownish to black [5] (Fig. 4e–h). Micrographs were taken with an Orthoplan microscope, equipped with a JVC TK-C1381EG digital color video camera.

### 2.6 Statistical analysis

Mean value±standard error (SE) were calculated for total cell counts and neutrophil, lymphocyte and macrophage differential count in BALF. The changes in cell and differential counts in BALF were compared with the *t* test between saline-treated and crocidolite-treated mice.
